# Combined Prognostic Value of the PROFUND Index and Serum Albumin for One-Year Mortality in Elderly Patients with Acute Heart Failure

**DOI:** 10.3390/jcm15093219

**Published:** 2026-04-23

**Authors:** Aladin Abdelhady Kishta Kishta, Marta M. Dolcet-Negre, María Jesús Rivas-López, Rocío García Alonso, Nuria Muñoz Rivas, Alicia Guzmán Carreras, Juan Igor Molina Puente, Manuel Méndez Bailón

**Affiliations:** 1 Departamento de Medicina Interna, Complejo Asistencial de Ávila, Avenida Juan Carlos I, s/n, 05071 Ávila, Spain; rociodoc.rg@gmail.com (R.G.A.); iggidoc@hotmail.com (J.I.M.P.); 2 Departamento de Estadística, Universidad de Salamanca, 37007 Salamanca, Spain; martamdn@usal.es (M.M.D.-N.); chusrl@usal.es (M.J.R.-L.); 3 Instituto de Física Fundamental y Matemáticas, Universidad de Salamanca, 37007 Salamanca, Spain; 4 Hospital Infanta Leonor, 28031 Madrid, Spain; nmrivas@hotmail.com; 5 Departamento de Medicina Interna, Hospital Clínico San Carlos, 28040 Madrid, Spainmanuelmenba@hotmail.com (M.M.B.)

**Keywords:** acute heart failure, multimorbidity, frailty, serum albumin, PROFUND index, biological reserve, prognostic models, risk stratification, internal medicine

## Abstract

**Background**: Older adults hospitalized with acute heart failure frequently present with multimorbidity, frailty, and reduced physiological reserve. This makes accurate prognostic assessment particularly challenging in internal medicine. Traditional heart failure risk models often fail to capture this multidimensional vulnerability. The PROFUND index, developed to estimate medium-term mortality in multimorbid patients, and serum albumin, an established biomarker of nutritional and inflammatory status, may provide complementary prognostic information. This formed the aim of the present study. This study’s objective is to evaluate the individual and combined prognostic value of the PROFUND index and serum albumin for one-year mortality in patients admitted with AHF. **Methods**: We conducted a prospective, multicenter cohort study within the PROFUNDIC registry. We included consecutive adults hospitalized with AHF or decompensated chronic heart failure who met European Society of Cardiology diagnostic criteria and had NT-proBNP levels > 1500 pg/mL. PROFUND scores were obtained at admission, and hypoalbuminaemia was dichotomized as ≤3.5 g/dL. The primary outcome was one-year mortality, analyzed using Kaplan–Meier survival estimates, Cox proportional hazards models, and time-dependent ROC curves. **Results**: Among 544 included patients (mean age 85 years; 60% women), high PROFUND scores (>7) were present in 39% and hypoalbuminaemia in 55%. Both variables independently predicted one-year mortality, with the highest risk observed in patients presenting both high PROFUND scores (HR 2.26; 95% CI 1.66–3.09; *p* < 0.001) and hypoalbuminaemia (HR 1.70; 95% CI 1.18–2.46; *p* = 0.0046). The combined use of these markers modestly improved discriminatory performance compared with the PROFUND index alone (HR 2.83; 95% CI 1.72–4.64; *p* < 0.000). **Conclusions**: These findings suggest that integrating clinical complexity, assessed by the PROFUND index, with serum albumin provides a simple and clinically meaningful approach to early risk stratification in very elderly multimorbid patients treated in internal medicine wards.

## 1. Introduction

Heart failure (HF) remains one of the most complex and resource-intensive conditions managed in internal medicine, particularly among older adults in whom multimorbidity, frailty, and functional dependency commonly converge. Also, acute heart failure (AHF) is one of the leading causes of hospitalization in older adults. It represents a growing challenge for healthcare systems in the context of population ageing, high comorbidity burden, and poor prognosis [1,2,3,4,5]. Despite advances in pharmacological and non-pharmacological therapies, improvements have not translated into proportional reductions in morbidity or mortality after acute heart failure episodes [6,7,8,9,10]. Patients admitted with AHF, particularly those managed in internal medicine wards, experience high early readmission rates, prolonged hospital stays, and elevated long-term mortality. These adverse outcomes cannot be fully explained by cardiac dysfunction alone [8,11]. Consequently, there is increasing recognition that prognostic tools must capture the multidimensional vulnerability of this population [2,12].

Traditional cardiology risk models are often derived from younger, more homogeneous cohorts with fewer comorbidities and greater physiological reserve than patients typically seen in internal medicine [1,3,13]. As a result, these models may underestimate risk in older adults burdened by multiple chronic conditions, cognitive impairment, social isolation, malnutrition, and limited resilience—factors that meaningfully influence outcomes but fall outside the scope of conventional heart failure scores [4,14,15,16]. In these patients, adverse outcomes result not only from HF severity but also from the cumulative burden of non-cardiac factors that limit recovery [2,3,12]. The central clinical challenge is to identify which components of a patient’s global health profile meaningfully modify prognosis and to integrate them into practical bedside tools.

Within this context, the PROFUND index is particularly relevant. Designed for multimorbid adults routinely managed in internal medicine, it integrates age, dependence, comorbidity burden, cognitive impairment, recent hospitalization, anemia, dyspnoea severity, and social support [13,14]. Together, these dimensions quantify global clinical vulnerability and provide a validated estimate of medium-term mortality in pluripathological populations [17,18,19]. Unlike disease-centred cardiology tools, the PROFUND index places heart failure within the broader context of frailty and chronic disease trajectories.

Serum albumin is a well-established prognostic marker in heart failure, yet it should not be interpreted as a pure indicator of nutritional status. Beyond its classical role as a nutritional indicator, albumin integrates physiological processes relevant to AHF evolution, including systemic inflammation, hepatic synthetic function, endothelial permeability, and intravascular oncotic pressure [20,21,22,23,24]. Hypoalbuminaemia has consistently been associated with reduced diuretic responsiveness, persistent congestion, increased vulnerability to infections, altered drug kinetics, and higher short- and long-term mortality [20,21,22,25,26]. In elderly patients, albumin therefore represents an integrative marker of biological resilience rather than a purely nutritional biomarker [22,24,26].

Beyond albumin alone, several nutrition-related prognostic tools have gained prominence in acute heart failure, particularly the Prognostic Nutritional Index (PNI), which incorporates serum albumin and lymphocyte count and has been associated with mortality, frailty, and adverse outcomes in AHF across multiple cohorts [27]. Although the PNI is informative, its applicability in real-world internal medicine settings may be limited by variability in lymphocyte counts during acute illness and by the multidimensional geriatric complexity seen in this population. In contrast, integrating serum albumin with a comprehensive multimorbidity frailty tool such as the PROFUND index may offer a more clinically coherent assessment within this specific demographic.

Importantly, the PROFUND index and serum albumin represent distinct but synergistic risk domains: PROFUND quantifies structural vulnerability stemming from chronic disease, functional decline, and social determinants, whereas albumin reflects immediate physiological reserve and acute resilience [12,14,16,19,20,21,22,24]. Their combination may therefore identify prognostic gradients that neither marker captures independently.

Given the high clinical heterogeneity of elderly AHF patients and the time-sensitive nature of decision-making in internal medicine [2,6,28], tools that are simple, reproducible, and available at admission are especially valuable. Albumin is universally measured on admission [20,21,22], and PROFUND relies on information already available from routine evaluation [15,18,19], making their combined use operationally feasible.

We hypothesized that integrating a measure of global clinical complexity with serum albumin would improve prognostic accuracy and provide clinically meaningful risk categories, thereby informing real-world decision-making in a vulnerable and underrepresented population [4,13,29,30].

## 2. Methods

This prospective, multicenter cohort study was conducted within the PROFUND IC registry, a national initiative led by the Spanish Society of Internal Medicine aimed at characterizing real-world outcomes in older patients hospitalized for acute heart failure (AHF). The registry reflects the pragmatic internal medicine setting, where acute cardiac decompensation often occurs alongside advanced comorbidity, functional impairment, and social vulnerability.

All consecutive adults admitted during the recruitment period with a primary diagnosis of AHF or acute decompensation of chronic heart failure were screened for inclusion. Eligibility followed European Society of Cardiology diagnostic criteria and required typical symptoms and signs of congestion (including progressive dyspnea, orthopnea, pulmonary crackles, jugular venous distension, hepatomegaly, ascites, or peripheral edema) together with objective evidence of cardiac dysfunction demonstrated by fulfilment of Framingham criteria and/or echocardiographic abnormalities. To improve diagnostic specificity in this elderly population, an admission NT-proBNP level > 1500 pg/mL was required. Deaths clearly attributable to non-cardiovascular causes were excluded to preserve internal validity. Informed consent was obtained from all participants prior to inclusion in the registry. In patients who were unable to provide informed consent due to cognitive impairment, acute clinical instability, or functional limitations, consent was obtained from a legally authorized representative or legal guardian, in accordance with national regulations and institutional ethical requirements.

Data were collected using standardized case report forms across all participating centres. In addition to demographic characteristics, cardiovascular risk factors, comorbidities, and chronic treatments. Data collection deliberately included geriatric and functional dimensions known to influence prognosis in internal medicine populations. Functional status was assessed using the Barthel Index, frailty using the Rockwood Clinical Frailty Scale, and nutritional status using the Mini Nutritional Assessment–Short Form. Overall comorbidity burden was quantified with the Charlson Comorbidity Index.

Missing data were handled using complete-case analysis for each model. The extent of missingness was limited and not considered sufficient to compromise model robustness; therefore, no imputation procedures were applied. Given the multicentre design, potential centre effects were explored descriptively. No hierarchical or random-effects modelling was prespecified because of the limited number of events per centre.

Multimorbidity-related risk was further summarized using the PROFUND index, a validated prognostic tool that integrates nine clinical and social domains, including age, functional dependence, cognitive impairment, recent hospitalizations, anemia, severe dyspnoea, and lack of caregiver support. Serum albumin concentrations were measured at admission according to local laboratory standards, and hypoalbuminaemia was defined a priori as ≤3.5 g/dL. Albumin was analyzed both independently and in combination with PROFUND risk strata to assess potential additive prognostic value.

Although PROFUND and serum albumin were also analyzed as continuous variables (Supplementary Material), primary analyses focused on predefined categories to enhance clinical applicability. This choice reflects the intended clinical application of the proposed approach, which aims to support early risk stratification rather than fine-grained individual risk prediction. In routine internal medicine practice, clinicians require pragmatic tools that allow patients to be classified into actionable risk groups, identifying those at very high risk who may benefit from intensified monitoring, anticipatory care planning, or early palliative discussions, versus those at lower risk in whom standard follow-up and resource allocation may be appropriate. Continuous modelling, while statistically informative, does not easily translate into bedside decision-making in complex, time-sensitive acute heart failure settings. Importantly, sensitivity analyses using continuous formulations of PROFUND and albumin yielded consistent associations with mortality, supporting the robustness of the findings and indicating that the observed prognostic gradients are not dependent on dichotomization (see Supplementary Material).

The primary endpoint was all-cause mortality within one-year of the index hospitalization. Follow-up was conducted through a systematic review of electronic health records within the PROFUND IC registry, supplemented by structured telephone contact with patients or caregivers when necessary. Time at risk was calculated from admission to death or censoring at 365 days.

Statistical analyses were performed using R (version 4.4.3; R Foundation for Statistical Computing, Vienna, Austria). Continuous variables were assessed for normality using the Shapiro–Wilk test and compared with analysis of variance or the Kruskal–Wallis test, as appropriate. Categorical variables were compared using chi-square or Fisher’s exact tests. Survival was analyzed using Kaplan–Meier methods with log-rank testing. Associations with mortality were examined using Cox proportional hazards models. Multivariable models included covariates selected a priori based on clinical judgement and prior evidence, including age, sex, comorbidity burden, functional status, renal function, and markers of heart failure severity; no univariable screening strategy was applied. Proportional hazards assumptions were verified using Schoenfeld residuals.

To complement survival analyses and account for censoring, time-dependent receiver operating characteristic (ROC) analyses were performed using the time*ROC* package in R. Discriminative performance was quantified by the area under the curve (AUC), sensitivity, specificity, and the Youden index at the 90th percentile of observed follow-up, a time point chosen to maximize event accrual while maintaining estimate stability.

Artificial intelligence tools (Microsoft 365 Copilot) were used exclusively to assist with language refinement and structural clarity. No AI tools were involved in data analysis, interpretation, or the generation of scientific conclusions.

## 3. Results

**Figure 1:**
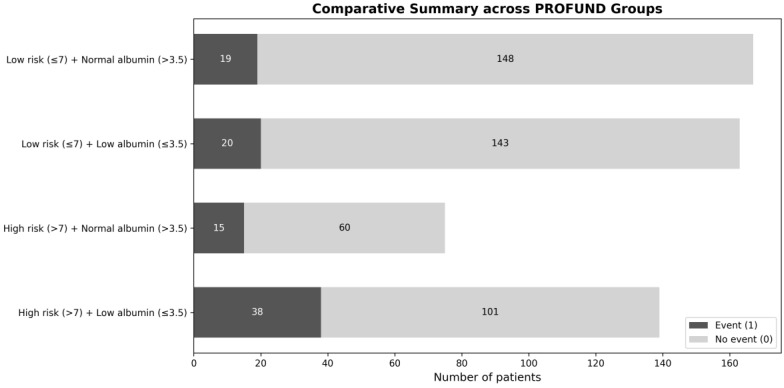
Flow diagram of patient selection and analytic cohort. Comparative distribution of clinical events across PROFUND-albumin risk categories. Bars represent the number of patients with and without the primary endpoint within each combined group: low PROFUND score (≤7) with normal albumin (>3.5 g/dL), low PROFUND score with hypoalbuminaemia (≤3.5 g/dL), high PROFUND score (>7) with normal albumin, and high PROFUND score with hypoalbuminaemia. Event (1) and non-event (0) frequencies are shown within each bar.

A total of 544 patients met the inclusion criteria and had complete data available for analysis (Figure 1), out of 913 individuals initially screened within the registry. The cohort reflected a typical internal medicine AHF population: very elderly patients (mean age 85 ± 6 years), with a predominance of women (59%). Multimorbidity and functional vulnerability were highly prevalent. Overall, 214 patients (39%) were classified as high risk according to the PROFUND index (>7), and 302 patients (55%) presented with hypoalbuminaemia (≤3.5 g/dL) at admission, underscoring the substantial burden of chronic complexity and impaired biological reserve (Table 1); a detailed table with treatment and secondary clinical variables has been moved to Supplementary Table S1 to improve readability and focus on prognostically relevant variables (Table 2).

**Figure 2:**
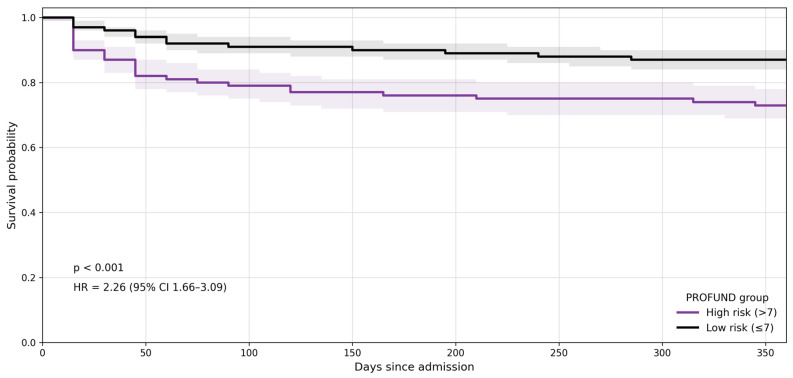
One-year all-cause mortality Kaplan–Meier survival curves according to PROFUND risk groups. High PROFUND was defined as >7. Kaplan–Meier survival curves according to PROFUND risk categories. Patients with high PROFUND scores (>7) exhibited significantly lower one-year survival compared with those in the low-risk group (≤7). Hazard ratio (HR) = 2.26 (95% CI 1.66–3.09); *p* < 0.001. Shaded areas represent 95% confidence intervals.

Survival analyses revealed a clear and graded association between increasing clinical complexity and one-year mortality. Patients classified as high risk according to the PROFUND index demonstrated significantly lower survival probabilities throughout the 12-month follow-up period compared with those in the low-risk group (Figure 2). In Cox proportional hazards models, a high PROFUND score was associated with more than a two-fold increase in mortality risk (HR 2.26; 95% CI 1.66–3.09; *p* < 0.001).

**Figure 3:**
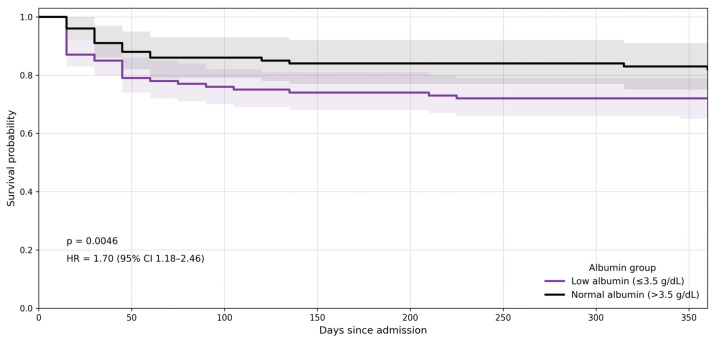
One-year all-cause mortality Kaplan–Meier survival curves according to serum albumin category. Hypoalbuminaemia defined as ≤3.5 g/dL. Kaplan–Meier survival curves stratified by serum albumin levels at admission. Patients with hypoalbuminaemia (≤3.5 g/dL) demonstrated reduced survival probability compared with those with normal albumin (>3.5 g/dL). Hazard ratio (HR) = 1.70 (95% CI 1.18–2.46); *p* = 0.0046. Shaded areas indicate 95% confidence intervals.

Similarly, serum albumin at admission demonstrated strong prognostic value. Patients with hypoalbuminaemia experienced significantly higher one-year mortality than those with preserved albumin levels, corresponding to a 70% relative increase in risk (HR 1.70; 95% CI 1.18–2.46; *p* = 0.0046) (Figure 3). Absolute mortality rates were consistently higher among patients with reduced albumin across both PROFUND strata, reinforcing the role of serum albumin in AHF prognosis.

**Figure 4:**
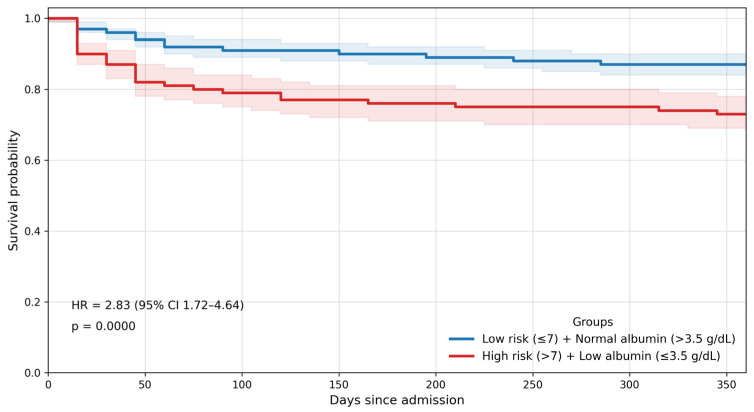
One-year all-cause mortality Kaplan–Meier survival analysis according to combined High PROFUND >7—Hypoalbuminaemia ≤3.5 g/dL and Reference group: low PROFUND (≤7)—normal albumin (>3.5 g/dL). Kaplan–Meier survival curves combining PROFUND risk category and albumin status. The group with a high PROFUND score (>7) and hypoalbuminaemia (≤3.5 g/dL) exhibited the lowest survival probability. Hazard ratio (HR) = 2.83 (95% CI 1.72–4.64); *p* = 0.0000. Shaded regions correspond to 95% confidence intervals.

When the PROFUND index and serum albumin were evaluated simultaneously, their prognostic effect was additive and clinically meaningful. Patients with both a high PROFUND score and hypoalbuminaemia constituted the highest-risk subgroup, exhibiting an almost three-fold increase in mortality risk compared with the reference category (low PROFUND score and normal albumin) (HR 2.83; 95% CI 1.72–4.64; *p* < 0.001) (Figure 4). This group also demonstrated the highest absolute event rates at one-year, highlighting the clinical relevance of combining structural vulnerability with serum albumin.

**Figure 5:**
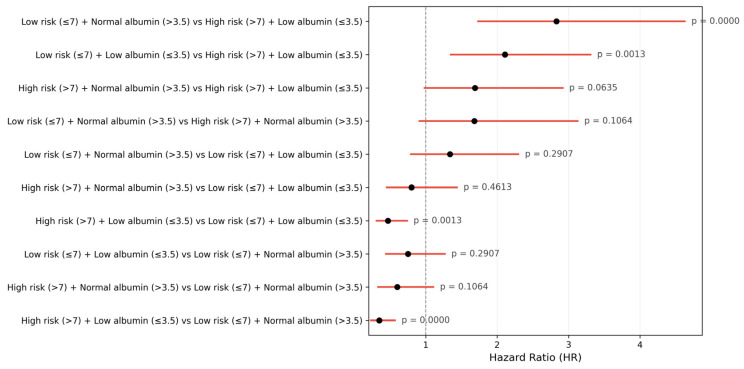
One-year all-cause mortality forest plot of hazard ratios for mortality across combined PROFUND–albumin categories derived from Cox proportional hazards models. Pairwise Cox proportional hazards comparisons across combined PROFUND and albumin groups. Hazard ratios with 95% confidence intervals are shown for each comparison, highlighting the additive prognostic effect of high multimorbidity burden (PROFUND > 7) and low albumin (≤3.5 g/dL). *p*-values are displayed for each contrast.

Subgroup analyses provided further insight into the interaction between multimorbidity and serum albumin. Among patients with high PROFUND scores, hypoalbuminaemia was associated with an increased risk of mortality, although the association narrowly failed to reach statistical significance (HR 1.69; 95% CI 0.97–2.93; *p* = 0.063). A similar directionally consistent, but non-significant, trend was observed among patients with low PROFUND scores (HR 1.34; 95% CI 0.78–2.31; *p* = 0.291) (Figure 5). These findings suggest that serum albumin modifies prognosis across the entire spectrum of multimorbidity severity.

**Figure 6:**
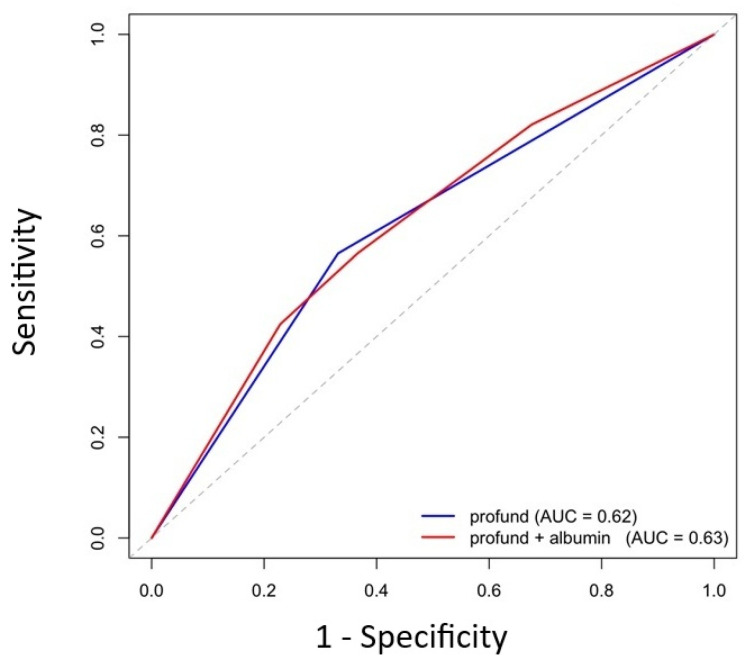
Time-dependent ROC curves comparing PROFUND alone versus combined PROFUND–albumin models at the 90th percentile of follow-up. Time-dependent ROC curves comparing the predictive performance of the PROFUND index alone versus the combined PROFUND + albumin model at 200 days. The combined model demonstrated slightly improved discrimination, with AUC = 0.63 compared with AUC = 0.62 for PROFUND alone.

Time-dependent ROC analysis showed that combining PROFUND with serum albumin yielded a numerically higher AUC than PROFUND alone (0.63 vs. 0.62), although the difference was minimal. Corresponding changes in sensitivity and specificity were modest, suggesting that the incremental prognostic gain of adding albumin was limited when assessed by global discrimination metrics. These findings indicate that the potential value of the combined approach lies more in clinical risk phenotyping than in meaningful improvements in AUC-based discrimination (Figure 6).

## 4. Discussion

In this prospective cohort of older, multimorbid adults hospitalized with AHF, we observed that the PROFUND index and serum albumin improved one-year mortality prediction when used together [13,15,18,19,20,21,22,23]. Each marker captured a distinct dimension of vulnerability: the PROFUND index quantified chronic clinical complexity [4,5,13,15,16], whereas serum albumin reflected biological reserve [20,21,22,23,24,25,26]. Their combined use enhanced risk discrimination beyond either marker individually.

Our results reinforce previous work demonstrating the value of the PROFUND index in internal medicine populations characterized by high multimorbidity and limited physiological reserve [13,15,18,19]. Patients with high PROFUND scores consistently exhibited elevated mortality risk, reflecting accumulated chronic disease, functional decline, and social fragility. In parallel, serum albumin provided additional prognostic information, but not as a pure nutritional marker. Instead, albumin represents an integrative biomarker influenced by systemic inflammation, hepatic synthetic function, venous congestion, endothelial permeability, and, only partly, nutritional status [19,20,21,22,23,24].

The interaction between multimorbidity and biological reserve was clinically relevant. While high PROFUND scores identified patients with substantial chronic vulnerability, preserved albumin levels appeared to mitigate part of this risk, suggesting that adequate physiological reserve may attenuate the adverse effects of accumulated complexity [13,15,18,19]. Conversely, hypoalbuminaemia identified biologically frail patients even within lower multimorbidity strata, highlighting that vulnerability in AHF is not fully captured by comorbidity counts alone [20,21,22,23,24,25]. This supports an integrative prognostic view in which outcomes result from the interaction between chronic disease burden, frailty, and acute systemic stress [4,8].

Emerging literature on nutrition-related prognostic tools in heart failure further contextualizes these findings. The Prognostic Nutritional Index (PNI), which incorporates serum albumin and lymphocyte count, has shown strong prognostic capacity in acute and chronic HF, correlating with frailty, malnutrition risk, and adverse outcomes [27]. While promising, the PNI may be less stable during acute decompensation due to fluctuations in inflammatory and immunological parameters. In contrast, pairing albumin with the PROFUND index incorporates both chronic vulnerability (multimorbidity, dependency, social determinants) and acute biological reserve, potentially offering a more pragmatic framework for real-world internal medicine settings, which differ significantly from typical cardiology cohorts.

Compared with widely used HF risk models such as the MAGGIC score, which perform well in broader HF populations, the combined PROFUND–albumin approach may better reflect geriatric complexity. The MAGGIC score was derived mainly from ambulatory or clinically stable cohorts and assumes heart failure to be the predominant driver of prognosis. It and other AHF scores often underestimate risk in older adults with cognitive impairment, frailty, or multisystem dysfunction—domains captured by PROFUND but absent from most cardiology-based tools. Thus, the integration of multimorbidity-driven and biology-driven markers may complement existing prognostic frameworks and improve calibration in elderly, highly heterogeneous AHF cohorts [31,32,33].

## 5. Clinical Implications

The combined assessment of PROFUND and albumin is feasible and operationally simple, as both measures are available within hours of admission and require no advanced technology. Identifying patients with a high PROFUND index, high multimorbidity, and low albumin may prompt:

- Intensified haemodynamic monitoring;
- Early nutritional and frailty assessment;
- Careful diuretic titration;
- Medication review and deprescribing;
- Proactive transitional care planning;
- Early palliative care involvement (in very high-risk profiles).

Using dichotomized thresholds facilitates rapid bedside decision-making and clinical translation. Although less granular than continuous modelling, these cut points reflect established risk categories already familiar to clinicians. This simplicity is particularly valuable given the frequent coexistence of polypharmacy, frailty, and cognitive impairment in this population.

Patients with high multimorbidity but preserved albumin may retain greater recovery potential and benefit from rehabilitation, medication optimization, and frailty-oriented interventions. Conversely, patients with low multimorbidity but hypoalbuminaemia may require targeted evaluation for potentially modifiable drivers such as inflammation, hepatic congestion, malnutrition, or sarcopenia.

Overall, combining PROFUND and albumin supports a more individualized, phenotype-tailored approach to AHF management that aligns with the principles of personalized medicine and the realities of internal medicine practice. Thereby offering a comprehensive strategy to refine risk stratification in older multimorbid patients with AHF.

## 6. Strengths and Limitations

This study has several strengths, including its real-world, multicentre design; its focus on this population, multimorbid with AHF patients, a population traditionally underrepresented in HF research; and the use of simple, widely available prognostic markers.

However, several limitations must be acknowledged:

- Observational design, which introduces potential residual confounding despite multivariable adjustment.
- Selection of patients from internal medicine wards, potentially limiting generalisability to cardiology units or younger HF populations.
- Unmeasured factors, including treatment changes during follow-up, specific HF therapies, and socioeconomic elements beyond caregiver availability, may have influenced outcomes.
- Dichotomisation of predictors, while clinically pragmatic, reduces granularity compared with continuous modelling.
- Lack of direct comparison with established models, such as MAGGIC, using head-to-head performance metrics in this specific population.

These limitations suggest that future research should validate our findings in independent cohorts, explore integration with existing heart failure scores, and assess whether combining multimorbidity indices with biological markers improves prognostic accuracy at scale.

## 7. Conclusions

The PROFUND index and serum albumin provide relevant prognostic information in older multimorbid adults hospitalized with acute heart failure. Their combination identifies patients at particularly high one-year mortality risk. As both measures are simple, inexpensive, and routinely obtained at admission, incorporating them into early evaluation processes can facilitate more personalized, anticipatory, and efficient care, supporting improved transitions and resource allocation in internal medicine settings.

## Figures and Tables

**Table 1:** Prevalence of multimorbidity and vulnerability domains according to PROFUND risk groups. This table presents the distribution of major multimorbidity conditions among patients with acute heart failure, comparing those in the low PROFUND risk group (≤7 points) with those in the high-risk group (>7 points). Reported conditions include cardiovascular, renal, respiratory, neurological, metabolic, autoimmune, hematologic, and oncologic comorbidities. Values are expressed as counts and percentages. Statistical comparisons were performed using chi-square or Fisher’s exact tests as appropriate.

	Level	Low Risk (≤7 Points) 330 Patients	High Risk (>7 Points) 214 Patients	Overall	*p*-Value
Heart failure (NYHA functional class II in clinical stability)	No	44 (11.4%)	34 (14.2%)	78 (12.5%)	0.3541
	Yes	343 (88.6%)	205 (85.8%)	548 (87.5%)	
Ischaemic heart disease	No	281 (72.6%)	167 (69.6%)	448 (71.5%)	0.4687
	Yes	106 (27.4%)	73 (30.4%)	179 (28.5%)	
Systemic autoimmune disease or vasculitis	No	371 (95.9%)	232 (96.7%)	603 (96.2%)	0.7687
	Yes	16 (4.1%)	8 (3.3%)	24 (3.8%)	
Chronic kidney disease	No	188 (48.6%)	103 (42.9%)	291 (46.4%)	0.1938
	Yes	199 (51.4%)	137 (57.1%)	336 (53.6%)	
Chronic respiratory disease	No	236 (61%)	152 (63.3%)	388 (61.9%)	0.6138
	Yes	151 (39%)	88 (36.7%)	239 (38.1%)	
Chronic inflammatory bowel disease	No	386 (99.7%)	235 (97.9%)	621 (99%)	0.0329
	Yes	1 (0.3%)	5 (2.1%)	6 (1%)	
Chronic liver disease	No	362 (93.5%)	229 (95.8%)	591 (94.4%)	0.3053
	Yes	25 (6.5%)	10 (4.2%)	35 (5.6%)	
History of stroke	No	329 (85.2%)	193 (80.4%)	522 (83.4%)	0.1433
	Yes	57 (14.8%)	47 (19.6%)	104 (16.6%)	
Neurological disease with permanent motor deficit	No	363 (93.8%)	215 (89.6%)	578 (92.2%)	0.0787
	Yes	24 (6.2%)	25 (10.4%)	49 (7.8%)	
Neurological disease with permanent cognitive impairment	No	368 (95.1%)	190 (79.2%)	558 (89%)	<0.001
	Yes	19 (4.9%)	50 (20.8%)	69 (11%)	
Symptomatic peripheral artery disease	No	354 (91.5%)	217 (90.4%)	571 (91.1%)	0.7591
	Yes	33 (8.5%)	23 (9.6%)	56 (8.9%)	
Diabetes mellitus with proliferative retinopathy and/or symptomatic neuropathy	No	323 (83.5%)	203 (84.6%)	526 (83.9%)	0.7954
	Yes	64 (16.5%)	37 (15.4%)	101 (16.1%)	
Chronic anaemia	No	266 (68.7%)	119 (49.6%)	385 (61.4%)	<0.001
	Yes	121 (31.3%)	121 (50.4%)	242 (38.6%)	
Active solid or haematological malignancy	No	369 (95.3%)	200 (83.3%)	569 (90.7%)	<0.001
	Yes	18 (4.7%)	40 (16.7%)	58 (9.3%)	
Chronic osteoarticular disease	No	270 (69.9%)	156 (65%)	426 (68.1%)	0.2290
	Yes	116 (30.1%)	84 (35%)	200 (31.9%)	

Values are presented as *n* (%). PROFUND = Prognostic Index for Patients with Multimorbidity. High PROFUND was defined as a score >7. Comparisons between groups were performed using the chi-square test or Fisher’s exact test, as appropriate. Overall refers to the total study population.

**Table 2:** Baseline characteristics according to PROFUND risk groups. This table presents the distribution of epidemiological characteristics, major comorbidities, clinical features, laboratory parameters, treatment-related variables, and functional or frailty-related measures in patients with acute heart failure, comparing those in the low-risk PROFUND group (≤7 points) with those in the high-risk group (>7 points). Data are presented as *n* (%), and between-group comparisons were performed using the chi-square or Fisher’s exact test, as appropriate. Additional clinical and treatment-related variables are provided in Supplementary Table S1.

Variable	Level	Low Risk (≤7 Points) 330 Patients	High Risk (>7 Points) 214 Patients	Overall	*p*-Value
Epidemiological Variables					
Sex	Male	159 (41.1%)	97 (40.4%)	256 (40.8%)	0.9347
	Female	228 (58.9%)	143 (59.6%)	371 (59.2%)	
Age	Years	387	240	627	
		84.4	88.1	85.9	<0.001
Body weight	kg	380	238	618	
		72.0	67.9	70.0	<0.001
Comorbidities					
NYHA functional class	I	30 (7.8%)	7 (2.9%)	37 (5.9%)	<0.001
	II	259 (66.9%)	72 (30%)	331 (52.8%)	
	III	86 (22.2%)	150 (62.5%)	236 (37.6%)	
	IV	12 (3.1%)	11 (4.6%)	23 (3.7%)	
Arterial hypertension	No	46 (11.9%)	19 (7.9%)	65 (10.4%)	0.147
	Yes	341 (88.1%)	221 (92.1%)	562 (89.6%)	
Diabetes mellitus	No	207 (53.5%)	135 (56.2%)	342 (54.5%)	0.5535
	Yes	180 (46.5%)	105 (43.8%)	285 (45.5%)	
Atrial fibrillation	No	109 (28.2%)	73 (30.4%)	182 (29%)	0.6078
	Yes	278 (71.8%)	167 (69.6%)	445 (71%)	
Chronic obstructive pulmonary disease	No	312 (80.6%)	199 (82.9%)	511 (81.5%)	0.5392
	Yes	75 (19.4%)	41 (17.1%)	116 (18.5%)	
Obstructive sleep apnoea syndrome	No	317 (81.9%)	201 (83.8%)	518 (82.6%)	0.6299
	Yes	70 (18.1%)	39 (16.2%)	109 (17.4%)	
Chronic kidney disease (eGFR * < 60 mL/min/1.73 m^2^)	No	202 (52.3%)	109 (45.4%)	311 (49.7%)	0.1095
	Yes	184 (47.7%)	131 (54.6%)	315 (50.3%)	
Dementia	No	374 (96.6%)	176 (73.3%)	550 (87.7%)	<0.001
	Yes	13 (3.4%)	64 (26.7%)	77 (12.3%)	
Active solid or haematological malignancy	No	382 (98.7%)	197 (82.1%)	579 (92.3%)	<0.001
	Yes	5 (1.3%)	43 (17.9%)	48 (7.7%)	
Analytical Variables					
Haemoglobin	g/dL	387	240	627	
		12.0	11.0	11.7	<0.001
Lymphocytes	Lymphocyte count	387	239	626	
		750.0	800.0	800.0	0.028
Serum albumin category	Low albumin (≤3.5 g/dL)	163 (49.4%)	139 (65%)	302 (55.5%)	<0.001
	Normal albumin (>3.5 g/dL)	167 (50.6%)	75 (35%)	242 (44.5%)	
Creatinine	mg/dL	387	240	627	
		1.2	1.2	1.2	0.022
Estimated glomerular filtration rate (CKD-EPI)	Numeric value mL/min/1.73 m^2^	387	240	627	
		47.5	42.1	44.7	<0.001
Total cholesterol	mg/dL	376	239	615	
		136.0	129.0	135.00	0.05
NT-proBNP	Numeric value pg/mL	386	240	626	
		5274.5	6423.0	5837.0	0.003
Cardiac troponin	Normal	233 (63.8%)	130 (56%)	363 (60.8%)	0.0692
	elevated	132 (36.2%)	102 (44%)	234 (39.2%)	
Prognostic variables					
Barthel Index	Index	387	240	627	
		90.0	55.0	80.0	<0.001
All-cause readmission within 12-months	Readmission within <12-months	136 (35.1%)	97 (40.4%)	233 (37.2%)	0.2137
	Without Readmission within <12-months	251 (64.9%)	143 (59.6%)	394 (62.8%)	
Death	Outcomes before 365-days	47 (12.1%)	63 (26.2%)	110 (17.5%)	<0.001
	Outcomes after 365-days (censored)	4 (1%)	1 (0.4%)	5 (0.8%)	
	Alive at 1-year (censored)	336 (86.8%)	176 (73.3%)	512 (81.7%)	

Values are presented as mean ± standard deviation, median [interquartile range], or *n* (%), as appropriate. Laboratory values were obtained at admission. Chronic kidney disease was defined as an estimated glomerular filtration rate <60 mL/min/1.73 m^2^ (CKD EPI equation). PROFUND = Prognostic Index for Patients with Multimorbidity. Comparisons between PROFUND groups were performed using Student’s *t*-test, the Mann–Whitney U test, the chi-square test, or Fisher’s exact test, as appropriate. A two-sided *p*-value < 0.05 was considered statistically significant. * Estimated glomerular filtration rate.

## Data Availability

Data supporting the findings of this study are available from the PROFUND-IC registry under institutional licence restrictions and are not publicly accessible. Data access requests may be considered by the Spanish Society of Internal Medicine.

## Supplementary Materials

The following supporting information can be downloaded at: https://www.mdpi.com/article/10.3390/jcm15093219/s1, Table S1. Baseline characteristics according to PROFUND risk groups.
