# Supplementary material for: Combined Prognostic Value of the PROFUND Index and Serum Albumin for One-Year Mortality in Elderly Patients with Acute Heart Failure
Source: J Clin Med. 2026 Apr 23;15(9):3219. doi: 10.3390/jcm15093219 (PMC13163894; doi:10.3390/jcm15093219)
Supplement: Supplementary file 1 [file jcm-15-03219-s001.zip › jcm-4205558-supplementary.pdf]

Table S1. Baseline characteristics according to PROFUND risk groups					
**Variable**	**Level**	**Low Risk (≤7 points) 330 patients**	**High Risk (>7 points) 214 patients**	**Overall**	**p-value**
**Epidemiological Variables**					
**Sex**	*Male*	159 (41.1%)	97 (40.4%)	256 (40.8%)	0.9347
	Female	228 (58.9%)	143 (59.6%)	371 (59.2%)	
**Age**	*Years*	*387*	*240*	*627*	
		84.4	88.1	85.9	**<0.001**
**Body weight**	*kg*	*380*	*238*	*618*	
		72.0	67.9	70.0	**<0.001**
**Height**	*cm*	*378*	*238*	*616*	
		160.0	160.0	160.0	0.242
**Comorbidities**					
**NYHA functional class**	*I*	30 (7.8%)	7 (2.9%)	37 (5.9%)	**<0.001**
	II	259 (66.9%)	72 (30%)	331 (52.8%)	
	III	86 (22.2%)	150 (62.5%)	236 (37.6%)	
	IV	12 (3.1%)	11 (4.6%)	23 (3.7%)	
**Arterial hypertension**	*No*	46 (11.9%)	19 (7.9%)	65 (10.4%)	0.147
	Yes	341 (88.1%)	221 (92.1%)	562 (89.6%)	
**Diabetes mellitus**	*No*	207 (53.5%)	135 (56.2%)	342 (54.5%)	0.5535
	Yes	180 (46.5%)	105 (43.8%)	285 (45.5%)	
**Atrial fibrillation**	*No*	109 (28.2%)	73 (30.4%)	182 (29%)	0.6078
	Yes	278 (71.8%)	167 (69.6%)	445 (71%)	
**Chronic obstructive pulmonary disease**	*No*	312 (80.6%)	199 (82.9%)	511 (81.5%)	0.5392
	Yes	75 (19.4%)	41 (17.1%)	116 (18.5%)	
**Obstructive sleep apnoea syndrome**	*No*	317 (81.9%)	201 (83.8%)	518 (82.6%)	0.6299
	Yes	70 (18.1%)	39 (16.2%)	109 (17.4%)	
**Chronic kidney disease (eGFR<60 mL/min/1.73 m²)**	*No*	202 (52.3%)	109 (45.4%)	311 (49.7%)	0.1095
	Yes	184 (47.7%)	131 (54.6%)	315 (50.3%)	
**Dementia**	*No*	374 (96.6%)	176 (73.3%)	550 (87.7%)	**<0.001**
	Yes	13 (3.4%)	64 (26.7%)	77 (12.3%)	
**Active solid or haematological malignancy**	*No*	382 (98.7%)	197 (82.1%)	579 (92.3%)	**<0.001**
	Yes	5 (1.3%)	43 (17.9%)	48 (7.7%)	
**Causes of Heart failure**	*Hypertensive*	147 (38%)	100 (41.7%)	247 (39.4%)	0.2412
	Ischaemic	73 (18.9%)	46 (19.2%)	119 (19%)	
	Toxic dilated cardiomyopathy	8 (2.1%)	2 (0.8%)	10 (1.6%)	
	Valvular	102 (26.4%)	50 (20.8%)	152 (24.2%)	
	Amyloidosis	8 (2.1%)	11 (4.6%)	19 (3%)	
	Other	49 (12.7%)	31 (12.9%)	80 (12.8%)	
**Clinical Variables**					
**Pulmonary crackles**	*No*	72 (18.6%)	26 (10.9%)	98 (15.7%)	**0.0065**
	Basal	225 (58.1%)	132 (55.2%)	357 (57%)	
	<50% of lung fields	53 (13.7%)	51 (21.3%)	104 (16.6%)	
	>50% of lung fields	37 (9.6%)	30 (12.6%)	67 (10.7%)	
**Delirium**	*No*	376 (97.2%)	181 (75.4%)	557 (88.8%)	**<0.001**
	Yes	11 (2.8%)	59 (24.6%)	70 (11.2%)	
**Dyspnea**	*No*	43 (11.2%)	12 (5%)	55 (8.8%)	**<0.001**
	Occasional	188 (48.8%)	93 (38.9%)	281 (45%)	
	Frequent	135 (35.1%)	116 (48.5%)	251 (40.2%)	
	Continuous	19 (4.9%)	18 (7.5%)	37 (5.9%)	

**Fatigue**	*No*	30 (7.8%)	13 (5.4%)	43 (6.9%)	**0.0027**
	Occasional	200 (51.9%)	94 (39.3%)	294 (47.1%)	
	Frequent	140 (36.4%)	115 (48.1%)	255 (40.9%)	
	Continuous	15 (3.9%)	17 (7.1%)	32 (5.1%)	
**Orthopnea**	*No*	138 (35.8%)	56 (23.4%)	194 (31.1%)	**0.0079**
	Occasional	148 (38.4%)	101 (42.3%)	249 (39.9%)	
	Frequent	78 (20.3%)	67 (28%)	145 (23.2%)	
	Continuous	21 (5.5%)	15 (6.3%)	36 (5.8%)	
**Low-cardiac-output symptoms**	*No*	356 (92.2%)	201 (83.8%)	557 (89%)	**0.0016**
	Yes	30 (7.8%)	39 (16.2%)	69 (11%)	
**Analytical Variables**					
**Haemoglobin**	*g/dL*	*387*	*240*	*627*	
		12.0	11.0	11.7	**<0.001**
**Lymphocytes**	*Lymphocyte count*	*387*	*239*	*626*	
		750.0	800.0	800.0	**0.028**
**Serum albumin category**	*Low albumin (≤3.5 g/dl)*	163 (49.4%)	139 (65%)	302 (55.5%)	**<0.001**
	Normal albumin (>3.5 g/dl)	167 (50.6%)	75 (35%)	242 (44.5%)	
**Creatinine**	*mg/dL*	*387*	*240*	*627*	
		1.2	1.2	1.2	**0.022**
**Estimated glomerular filtration rate (CKD-EPI)**	*Numeric value*	*387*	*240*	*627*	
	mL/min/1.73 m²	47.5	42.1	44.7	**<0.001**
**Total cholesterol**	*mg/dL*	*376*	*239*	*615*	
		136.0	129.0	135.00	**0.05**
**NT-proBNP**	*Numeric value pg/ml*	*386*	*240*	*626*	
		5274.5	6423.0	5837.0	**0.003**
**Cardiac troponin**	*Normal*	233 (63.8%)	130 (56%)	363 (60.8%)	0.0692
	elevated	132 (36.2%)	102 (44%)	234 (39.2%)	
**Treatment**					
**Beta-blocker**	*No*	136 (35.2%)	104 (43.9%)	240 (38.5%)	**0.0386**
	Yes	250 (64.8%)	133 (56.1%)	383 (61.5%)	
**Diuretics**	*No*	24 (6.2%)	27 (11.4%)	51 (8.2%)	0.1208
	Furosemide	315 (81.6%)	186 (78.8%)	501 (80.5%)	
	Hydrochlorothiazide	5 (1.3%)	2 (0.8%)	7 (1.1%)	
	Furosemide + Hydrochlorothiazide	42 (10.9%)	21 (8.9%)	63 (10.1%)	
**Mineralocorticoid receptor antagonists**	*No*	234 (61.1%)	160 (67.8%)	394 (63.7%)	0.2384
	Spironolactone	126 (32.9%)	65 (27.5%)	191 (30.9%)	
	Eplerenone	23 (6%)	11 (4.7%)	34 (5.5%)	
**Angiotensin-Converting Enzyme inhibitors**	*No*	297 (76.9%)	197 (83.1%)	494 (79.3%)	0.0808
	Yes	89 (23.1%)	40 (16.9%)	129 (20.7%)	
**Angiotensin II receptor blockers**	*No*	294 (76.4%)	190 (80.2%)	484 (77.8%)	0.3126
	Yes	91 (23.6%)	47 (19.8%)	138 (22.2%)	
**Angiotensin receptor-neprilysin inhibitor**	*No*	353 (91.7%)	214 (90.3%)	567 (91.2%)	0.6535
	Yes	32 (8.3%)	23 (9.7%)	55 (8.8%)	
**Sodium-glucose co-transporter 2 inhibitors**	*Empagliflozin*	68 (50%)	34 (49.3%)	102 (49.8%)	1.0000
	Dapagliflozin	67 (49.3%)	34 (49.3%)	101 (49.3%)	
	Other	1 (0.7%)	1 (1.4%)	2 (1%)	
**Lipid-lowering therapy**	*Statin*	150 (86.2%)	84 (88.4%)	234 (87%)	0.8438
	Ezetimibe	4 (2.3%)	1 (1.1%)	5 (1.9%)	
	Statin + Ezetimibe	20 (11.5%)	10 (10.5%)	30 (11.2%)	
**Anticoagulation type**	*Vitamin K Antagonists*	87 (39.5%)	46 (36.8%)	133 (38.6%)	0.9635

	Dabigatran	7 (3.2%)	3 (2.4%)	10 (2.9%)	
	Rivaroxaban	25 (11.4%)	16 (12.8%)	41 (11.9%)	
	Apixaban	71 (32.3%)	41 (32.8%)	112 (32.5%)	
	Edoxaban	30 (13.6%)	19 (15.2%)	49 (14.2%)	
**Protein supplements**	*No*	362 (93.8%)	209 (87.8%)	571 (91.5%)	**0.0143**
	Yes	24 (6.2%)	29 (12.2%)	53 (8.5%)	
**Intravenous iron therapy**	*No*	270 (70.1%)	163 (68.5%)	433 (69.5%)	0.7315
	Yes	115 (29.9%)	75 (31.5%)	190 (30.5%)	
**Number of prescribed medications**	*Numeric value*	378	233	611	
		10.0	11.0	11.0	0.1280
**Prognostic variables**					
**Barthel Index**	*Index*	*387*	*240*	*627*	
		90.0	55.0	80.0	**<0.001**
**All-cause readmission within 12 months**	*Readmission within <12 months*	136 (35.1%)	97 (40.4%)	233 (37.2%)	0.2137
	Without Readmission within <12 months	251 (64.9%)	143 (59.6%)	394 (62.8%)	
**Death**	*Outcomes before 365 days*	47 (12.1%)	63 (26.2%)	110 (17.5%)	**<0.001**
	Outcomes after 365 days (censored)	4 (1%)	1 (0.4%)	5 (0.8%)	
	Alive at 1 year (censored)	336 (86.8%)	176 (73.3%)	512 (81.7%)	

Values are presented as mean ± standard deviation, median [interquartile range], or n (%), as appropriate. Laboratory values were obtained at admission. Chronic kidney disease was defined as an estimated glomerular filtration rate <60 mL/min/1.73 m² (CKD EPI equation). PROFUND = Prognostic Index for Patients with Multimorbidity. Comparisons between PROFUND groups were performed using Student’s t test, Mann–Whitney U test, Chi square test, or Fisher’s exact test, as appropriate. A two sided p value <0.05 was considered statistically significant.
